# Proteasome Complexes and Their Heterogeneity in Colorectal, Breast and Pancreatic Cancers

**DOI:** 10.7150/jca.52414

**Published:** 2021-03-05

**Authors:** Diana Zagirova, Rebecca Autenried, Morgan E. Nelson, Khosrow Rezvani

**Affiliations:** Division of Basic Biomedical Sciences, Sanford School of Medicine, University of South Dakota, 414 E. Clark Street, Lee Medical Building, Vermillion, SD 57069, USA.

**Keywords:** proteasome, colon, breast, pancreas, cancer, bortezomib

## Abstract

Targeting the ubiquitin-proteasome system (UPS) - in particular, the proteasome complex - has emerged as an attractive novel cancer therapy. While several proteasome inhibitors have been successfully approved by the Food and Drug Administration for the treatment of hematological malignancies, the clinical efficacy of these inhibitors is unexpectedly lower in the treatment of solid tumors due to the functional and structural heterogeneity of proteasomes in solid tumors. There are ongoing trials to examine the effectiveness of compound and novel proteasome inhibitors that can target solid tumors either alone or in combination with conventional chemotherapeutic agents. The modest therapeutic efficacy of proteasome inhibitors such as bortezomib in solid malignancies demands further research to clarify the exact effects of these proteasome inhibitors on different proteasomes present in cancer cells. The structural, cellular localization and functional analysis of the proteasome complexes in solid tumors originated from different tissues provides new insights into the diversity of proteasomes' responses to inhibitors. In this study, we used an optimized iodixanol gradient ultracentrifugation to purify a native form of proteasome complexes with their intact associated protein partners enriched within distinct cellular compartments. It is therefore possible to isolate proteasome subcomplexes with far greater resolution than sucrose or glycerol fractionations. We have identified differences in the catalytic activities, subcellular distribution, and inhibitor sensitivity of cytoplasmic proteasomes isolated from human colon, breast, and pancreatic cancer cell lines. Our developed techniques and generated results will serve as a valuable guideline for investigators developing a new generation of proteasome inhibitors as an effective targeted therapy for solid tumors.

## Introduction

Remaining the first-choice approach for cancer treatment, the application of chemotherapy is significantly constrained by an intrinsic or acquired resistance associated with most solid tumors 1 and a wide range of side effects 2. The limitations associated with traditional chemotherapies have led to the development of targeted cancer therapies that interfere with specific signaling pathways dysregulated in tumors 3. Blocking a key protein or pathway responsible for the initiation, development, or maintenance of tissue-specific tumors is an effective approach to cancer-targeted therapy 4.

Inhibition of the ubiquitin-proteasome system (UPS) became a promising strategy due to the dependence of tumor metabolism and survival on a functional UPS 5-7. The deregulated proteasome complex and its protein partners lead to the upregulation of cell proliferation 8, 9 while simultaneously downregulating apoptosis 10-12. Together, these roles make the UPS an emerging target in both early- and late-stage cancer therapeutics development 7.

Bortezomib is a first-generation proteasome inhibitor that has received Food and Drug Administration (FDA) approval for the treatment of multiple myeloma 13 and mantle cell lymphoma 14. The second generation of proteasome inhibitors was aimed at addressing the limitations of bortezomib connected with off-target activity and acquired resistance by targeting multiple β catalytic subunits in an irreversible manner alongside enhanced oral availability 15. Over the past decade, several new small molecules that influence various essential components of the UPS have been reported 7.

Although many proteasome inhibitors have been successfully applied as anti-cancer agents, a set of complications has arisen with the development of cancer drugs targeting the UPS. These include limited therapeutic effects in solid tumors and acquired resistance after the prescription of UPS inhibitors 16-19. A better understanding of the factors causing proteasome sensitivity or resistance to current and novel inhibitors will increase the clinical efficacy of novel therapeutic approaches 20.

The aim of this current study was twofold: 1) to introduce an alternative method capable of isolating proteasome complexes in their physiological conditions from cell and tissue lysates. 2) to validate whether the existence of multiple proteasome subpopulations with different levels of response to proteasome inhibitors is mechanistically responsible for the partial/variable effectiveness of proteasome inhibitors in solid tumors. In this context, we identified the baseline catalytic activity and inhibitor sensitivity of proteasome complexes in human breast, colon, and pancreatic cancer cell lines as well as a set of non-cancerous human cell lines. We used an optimized iodixanol gradient ultracentrifugation procedure to physiologically isolate proteasome complexes in a subcellular-dependent manner. To confirm the integrity of the iodixanol fractionation approach, a set of western blot and enzymatic assays were conducted to verify co-sedimentation of known proteins associated with proteasome complexes as well as colocalization with intact endoplasmic reticulum (ER) and Golgi compartments. Using the biochemical assay screen, we measured the chymotrypsin-, trypsin-, and caspase-like catalytic activities of the proteasome in collected fractions. The results show a set of subclasses of proteasome complexes among cancer cell lines originated from different tissue origins. Moreover, fractionation of the subpopulation of proteasome complexes revealed the presence of proteasome complexes with different sensitivities to proteasome inhibitors in breast, colon, and pancreatic cancers based on specific proteasome catalytic activities. The presence of multiple proteasome subpopulations with heterogeneity in response to proteasome inhibitors, particularly bortezomib, potentially explains the diverse and modest responses of selective solid tumors to proteasome inhibitors. The results and optimized technique for intact proteasome extraction presented in this study open a new direction for optimizing diagnostic assays and the more effective proteasome inhibitors in several common solid tumors with insufficient response to conventional chemotherapies.

## Materials and Methods

### Cell culture and protein extraction

Human HEK-293, CCD-18Co (CCD-18), HCT-116, T84, MCF7, MIA PaCa-2, and HPAFII cancer cells provided by the ATCC (American Type Culture Collection, Manassas, VA, USA) were cultured per ATCC instructions. CCD-18Co is a non-cancer human colon fibroblast cell line. HCT-116 is a primary colorectal carcinoma cell line and T84 is a colon cancer cell line derived from lung tissues as the metastatic site. MCF7 is a breast cancer cell line derived from pleural effusion as the metastatic site. The HPAF-II cell line is a well-differentiated human pancreatic carcinoma cell line, while MiaPaCa-2 is a poorly differentiated pancreatic cell line (Table S1) 21. Cells around 70% confluency were harvested via mechanical scraping at 4 °C. Cytoplasmic and nucleus proteins were isolated using the NE-PER nuclear and cytoplasmic protein extraction kit (Thermo Fisher Scientific, Grand Island, NY, USA) in the presence of 2 mM ATP (Adenosine 5'-triphosphate, catalog #A2383, Sigma-Aldrich, St. Louis, MO, USA) as previously recommended 22. The total protein concentration was measured using the Pierce BCA protein assay (Thermo Fisher Scientific).

### Glycerol gradient ultracentrifugation

A homogenizing buffer was prepared by combining 1.2 ml of 100 mM ATP (pH 7.5), 1.2 ml of 50 mM DTT, 6 ml of 50 mM MgCl_2_, 2.4ml of 500 mM Tris (pH 7.5), 49.2 ml of H_2_O, and 4 ml of pure glycerol. 2.5 ml volumes of 10%, 20%, 30%, and 40% glycerol each were layered into Beckman Ultra-Clear 13.2 ml centrifuge tubes using a Labconco Auto Densi-Flow peristaltic pump (Labconco, Kansas City, MO, USA). Cytoplasmic or nuclear lysates were loaded onto the discontinuous linear gradient. The samples were spun at 28,500 rpm (100 000 *g*) for 18 hours at 4°C using a Beckman SW41Ti rotor at 4 °C (Beckman Coulter, Brea, CA, USA). Following centrifugation, the gradient became continuous with protein complexes distributed according to their sizes and compositions. Twenty fractions of 500 µl were collected using a Labconco Auto Densi-Flow peristaltic pump and stored at -20 °C. The density of each fraction was determined by weighing an equal volume per fraction using an analytical balance.

### Iodixanol gradient ultracentrifugation

**Figure 1:**
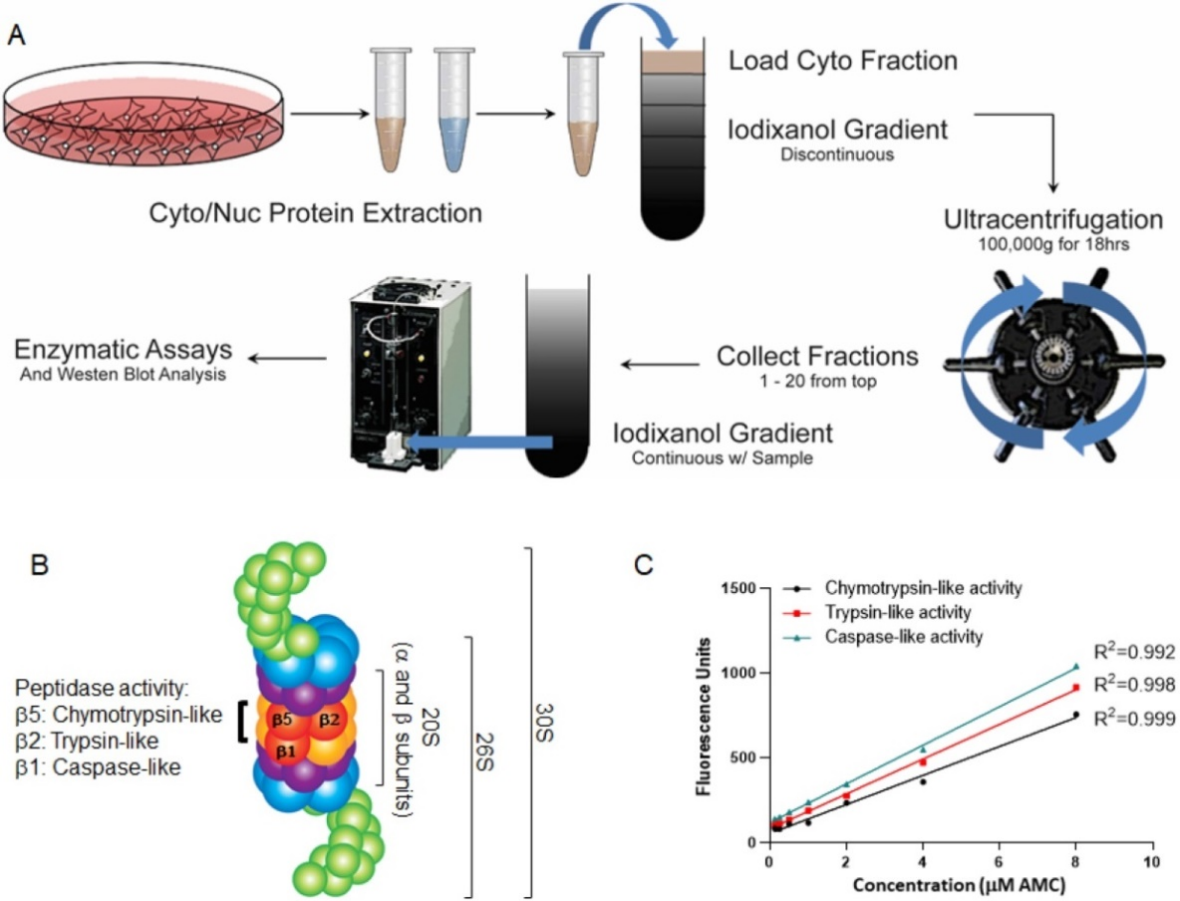
** Experimental design of proteasome study.** (A) Overview of the experimental study. Cytoplasmic and nuclear lysates were subjected to an iodixanol gradient fractionation. Twenty fractions were collected and analyzed for proteasomal catalytic activities with and without proteasome inhibitors. (B) Structure of the 20S, 26S, and 30S proteasomes. The proteasome can be composed of three particles: one is the catalytic core or 20S particle (yellow and orange); for the other two, the 19S regulatory particle consists of 19 individual proteins divided into a 10-protein 'base' (blue) subassembly and a 9-protein 'lid' sub-particle (green), generating either 26S (one 19S cap) or 30S (two 19S caps). (C) Graphs show the linear correlation between fluorescence intensity and the amount of substrate-AMC-hydrolyzing activity in reaction, plotted as arbitrary fluorescence units (AFU). The fluorescence intensity was measured at 380 nm excitation and 460 nm minus background fluorescence.

With the cytoplasmic fractions, we performed isopycnic iodixanol gradient subcellular fractionation as previously described 23. To prepare a 50% (w/v) iodixanol working solution, we added five volumes of OptiPrep (Cosmo Bio USA, Carlsbad, CA, USA) to 1 vol of 0.25 M sucrose, 6 mM EDTA, 60 mM HEPES, pH 7.4. OptiPrep™ is a 60% (w/v) solution of iodixanol in water, density = 1.32 g/ml. Following the steps previously described 24 and associated protocols on the Axis-Shield website (http://www.axis-shield-density-gradient-media.com/), we mixed a certain ratio of homogenization medium (a HEPES based buffer [10 mM, pH 7.4] with 0.25 M sucrose, 1 mM EDTA, and 2 mM ATP) with certain volumes of iodixanol working solution to prepare the 2.5 ml gradient dilutions of 8%, 16%, 28%, and 38% each of iodixanol (supplement protocol 1). The osmolality of these dilutions is in the range of 295-310 mOsm. We layered 2.5 ml of each dilution into Beckman Ultra-Clear 13.2ml centrifuge tubes. Cytoplasmic lysates were carefully loaded onto the discontinuous gradient and, using an SW41Ti rotor and a Beckman L8-55 ultracentrifuge, the samples were centrifuged at 28,500 rpm (100 000 *g*) for 18 hours at 4 °C. Eighteen to twenty fractions (approximately 500 µl) were collected using a Labconco Auto Densi-Flow peristaltic pump and stored at -20 °C (Fig. 1A). Fraction densities were determined by weighing a known volume using an analytical balance.

### Assaying protease activity in cell extracts

We conducted an AMC/substrate calibration assay followed by a proteasome assay for three enzymatic activities of pure 20S proteasome according to the instructions provided by the manufacturer (Enzo Life Sciences, Farmingdale, NY, USA). Fluorogenic substrates Suc-LLVY-AMC, Bz-Val-Gly-Arg-AMC, and Z-LLE-AMC were used to determine the chymotrypsin-like (β5), trypsin-like (β2), and caspase-like (peptidylglutamyl-peptide hydrolyzing-β1) activities of the 20S proteasome (Fig. 1B). Figure 1C shows a linear correlation between the fluorescence and the amount of AMC released in reaction presented by the arbitrary fluorescence unit (AFU) using a plate reader at excitation/emission wavelengths of 380 nm/460 nm, respectively.

To measure the three proteasomal activities in collected fractions, an assay buffer was prepared containing 50 mM Tris-HCl, 40 mM KCl, and 5 mM MgCl2 and adjusted to a pH of 7.5. Immediately prior to the assay, 50 µl of 100 mM ATP, 10 µl of 1 M DTT, and 100 µl of 50 mg/ml BSA were added to the buffer at 4 °C 22. Proteasome activity was analyzed using fluorescent substrates specific to the chymotrypsin-like, caspase-like, and trypsin-like activities. Arbitrary Fluorescence Units (AFU) were determined based on cleaved AMC fluorescence for each fraction using a Perkin Elmer 2030 Victor X2 fluorometer set to 380 nm excitation and 460 nm absorption at a time point of 5 hours. We conducted all the iodixanol gradient fractionation assays in the presence of 2 mM ATP to avoid dissociation of the 19S cap from the 20S proteasome core (Fig. 1B) 25. Because ATP is stable for several hours at 4 °C, the first measurement of proteasomal catalytic activity was taken 5 hours after substrate addition where we obtained maximum proteasomal activities. We saw a linear elevation of the activity or minimal changes from the initial peaks over a 48-hour time period.

### Analysis of proteasome catalytic activity

As previously recommended, to compare proteasome activity in different samples we loaded equal amounts of total protein (~8.6 mg total cell lysate) for all iodixanol fractionations 22. Proteasome-specific activity was determined by subtracting proteolytic activity in the presence of bortezomib from total proteolytic activity. Activity assay data were analyzed as follows: The AFU values of three repeats of chymotrypsin-, trypsin-, and caspase-like activity assays were averaged, and from this value was subtracted the AFU of a blank well. The proteolytic activity of each fraction in the presence of the proteasome inhibitor bortezomib (10 nM; final concentration in 200 µl of proteasome buffer per well) was also measured and it was considered non-proteasomal catalytic activity. These non-proteasomal catalytic activity values were subtracted from the chymotrypsin-, trypsin-, and caspase-AFU values to yield pure proteasome activity data, as previously described 26. AFU data was then graphed as a percent of total enzymatic activity (chymotrypsin-, trypsin-, or caspase-like activities), which allowed for comparison between cell lines. Normal and cancerous cell lines were compared based on proteasomal catalytic activities and their responses to proteasome inhibitors. In addition to bortezomib, collected fractions were treated with MG132 proteasome inhibitor (100 nM; final concentration in 200 µl of proteasome buffer per well, for all except trypsin-like assays) or TLCK proteasome inhibitor (100 nM; final concentration in 200 µl of proteasome buffer per well, only for trypsin-like assays).

### Endoplasmic reticulum and Golgi apparatus assays

Assays were run on the collected fractions to determine the sedimented locations of the endoplasmic reticulum (ER) and Golgi compartments using arylesterase and L-leucine-(4-methyl-7-coumarinylamide) hydrochloride assays, respectively 27. Assays served to determine the locations of intact ER and Golgi compartments and confirm the efficiency of the iodixanol gradient fractionations due to the consistent detection of the ER and Golgi within expected fractions, as previously described 28.

### Western blot and antibodies

Collected fractions were subjected to 4-12% SDS-PAGE, transferred to nitrocellulose membranes (iBlot system, Thermo Fisher Scientific), and probed with antibodies to locate the 20S and 19S particles. We used pan-alpha antibody (proteasome 20S α1,2,3,5,6,&7 subunits, mAb MCP231, 1:1000, Enzo Life Sciences) and β5 antibody to locate the 20S complex, and the Rpt6/S8 antibody (proteasome 19S ATPase subunit Rpt6, mAb p45-110, 1:500 to 1:1000, Enzo Life Sciences) to locate the 19S cap. Primary antibodies p47 (NSFL1, 1:2000, Abcam, Cambridge, MA, USA), pan-14-3-3 (1:1000, Santa Cruz, Dallas, USA), Gankyrin (1:500, Santa Cruz), and p97 (1:1000, Abcam) were used to confirm the association of the proteasome complex with its standard partner proteins. IRDye infrared secondary antibodies (IRDye 800CW and IRDye 680RD) and an Odyssey® CLx Imaging System (LI-COR Biosciences, Lincoln, NE, USA) were used to visualize the protein signals. We could not use an 18-well gel because wells in those longer gels have lower volume capacity (BIORAD, 4-15% Criterion™ TGX™ Precast Midi Protein Gel, 18 wells, 30 µl #5671084) as compared to our 10-well SDS-PAGE gel (ThermoFisher, Tris-Glycine Gels, 10 wells, 60 µl. #XP00100BOX). To apply the same treatment for both sides, we purposefully kept the intensity of exposure the same for all membranes in Figures 5 and 6 using LI-COR technology. Of course, due to the slight differences between individual SDS-PAGE gels and nitrocellulose membranes, we saw some differences in terms of brightness between the two sides. Despite differences in brightness, the WB panels in Figures 5 and 6 were able to confirm the accuracy of proteasome activities recorded and illustrated in the same figures.

### Statistical analysis

Enzymatic assay experiments in HEK-293 cells were done in triplicate for each glycerol and iodixanol gradient with the average values shown. The method was optimized in HEK-293 cells to obtain results for the proteasomal activities in cancer cell lines HCT-116, MCF7, HPAFII, and MIA PaCa-2 as well as the human colon fibroblast cell line CCD-18. Catalytic assays (three activities of the proteasome) and enzymatic assay experiments (ER and Golgi assays) were done in triplicate wells and repeated two separate times with similar results. All diagrams were generated by using GraphPad Prism 8.0 (GraphPad Software, San Diego, CA, USA).

## Results

### Distribution of proteasome complexes in glycerol gradient versus iodixanol gradient fractionation

**Figure 2:**
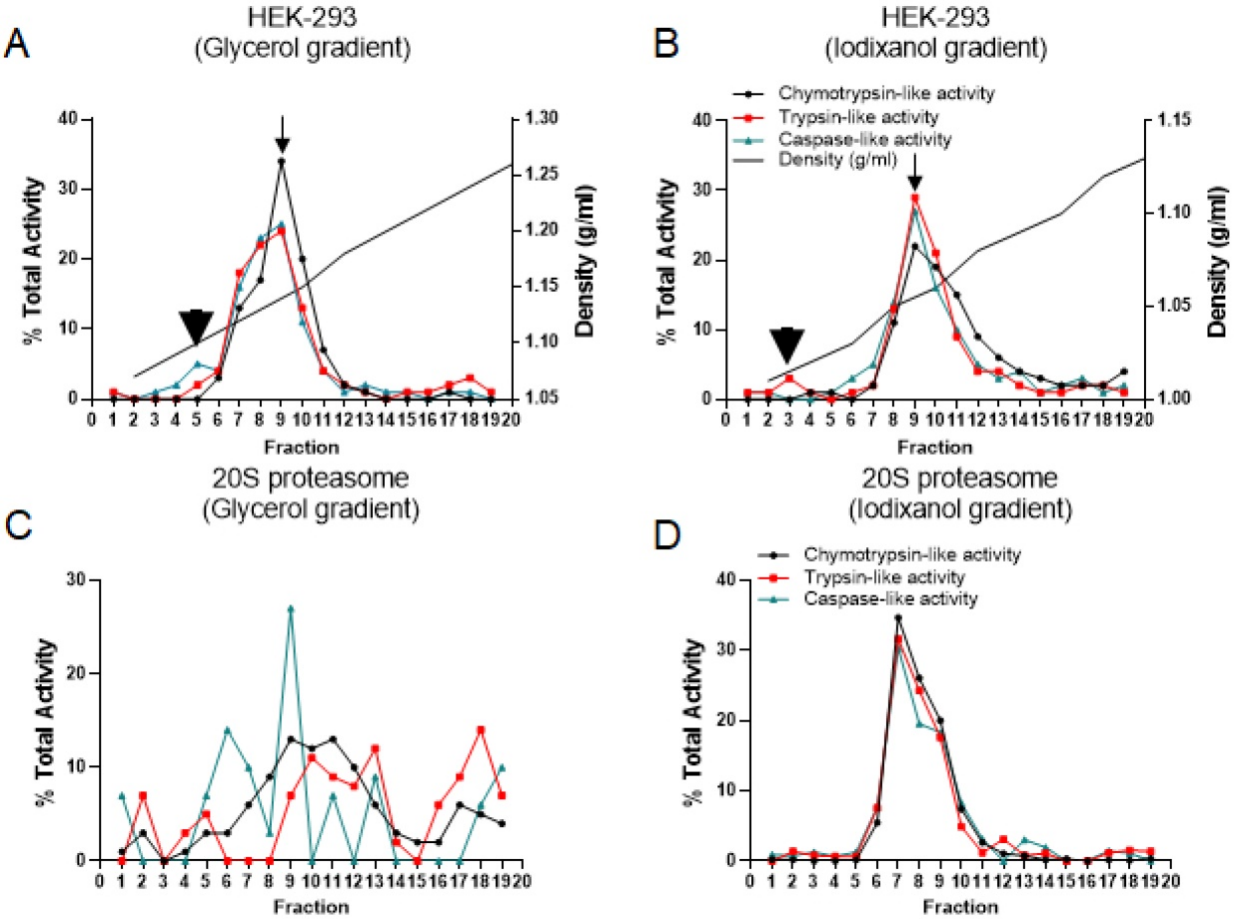
** Distribution of proteasome complexes in glycerol gradient versus iodixanol gradient fractionation**. Glycerol (A, C) and iodixanol (B, D) density gradient fractionations were conducted on the HEK-293 cytoplasmic cell lysates (A, B) and purified proteasomal 20S (C, D). Proteasome chymotrypsin-, trypsin-, and caspase-like activities in the fractions collected were assessed, with the measurement of AMC fluorescence specific for each activity. Arrowheads show the potential 20S proteasome catalytic activity (fraction 5, Panel A; fraction 3, Panel B), and arrows show the peak of three potential 26S proteasome catalytic activities (fraction 9). The experiments indicate that iodixanol gradient fractionation maintains proteasome integrity and allows effective isolation of the proteasome complex as holoenzyme or isolated subcomplexes.

It has been reported that iodixanol as an aqueous solution is iso-osmotic and therefore can preserve intracellular compartments such as Golgi and ER as well as their associated protein complexes in their physiological conditions 23, 29, 30. The proteasome complexes and their non-proteasomal partners 31 are free complexes in the cytoplasm or associated with different cellular compartments. In comparison to traditional glycerol and sucrose fractionation (Table S2), iodixanol offers an effective strategy for the study of proteasome complexes and their subpopulations 32 as well as their subcellular localizations 33, 34 in their native forms. To compare the glycerol and iodixanol fractionation methodologies, the HEK-293 cytoplasmic cell lysates and purified 20S proteasome (gift of Dr. George DeMartino, UT Southwestern) were subjected to glycerol and iodixanol gradient fractions (Fig. 2). While the percent of average propteasomal activities recorded in fraction 9 was similar for both methods, 26% for glycerol and 28% for iodixanol, the distribution of proteasome catalytic activities showed differences in all other fractions, suggesting a different migration pattern through the gradient. Both size and composition of protein complexes are critical parameters for the migration differences between glycerol and iodixanol gradient fractionations 35. In the glycerol fractionation, the three different proteasomal catalytic activities showed a slight shift. However, the three proteasome activities showed the same single peak (Fig. 2, Panels A and B; arrows at fraction 9), which likely corresponds to the 26S proteasome complexes. Additionally, the 20S proteasome catalytic activity has been mixed with 26S in the glycerol gradient fractionation, while the iodixanol gradient showed a distinct peak for the potential 20S complex (Fig. 2, Panel A versus Panel B; arrowheads). Finally, the density of collected fractions (g/ml) showed that fraction 9 had a density of ~1.14g/ml in the glycerol gradient fractions (Fig. 2A) while the same fraction in the iodixanol gradient had a density of ~1.06 g/ml (Fig. 2B).

To further compare the separating efficiency of the iodixanol gradient versus the glycerol gradient, a purified form of proteasomal 20S was fractionated by glycerol and iodixanol gradients (Fig. 2, Panels C and D). Proteasome catalytic assays were performed in order to assess fractions of proteasome sedimentation as well as determine the ability of the gradients to maintain proteasome functionality. In the iodixanol gradient, pure 20S sedimented into fractions 6 through 10, peaking in fraction 7 (Fig. 2D). In the glycerol gradient, the control 20S peaked with caspase-like activity in fraction 9 and with chymotrypsin and trypsin activity in fractions 9 and 11 (Fig. 2C). However, the 20S proteasome did not show the clear sedimentation pattern observed in the iodixanol gradient (Fig. 2D). Thus, analysis of the two fractionation methods using the HEK-293 cell line and pure 20S proteasome shows that iodixanol ultracentrifugation maintains proteasome functionality and allows the achievement of a more relevant pattern of the distribution per proteasomal activities. The proteolytic activities illustrated in Figure 2 as percentages correspond to the total percentage of proteasomal activities suppressed by bortezomib (see “Materials and Methods” section).

### Measurement of proteasome activities shows different proteasome profiles in the nuclear and cytoplasmic compartments

It has already been shown that the proteasome complex, with its multi-catalytic machinery, targets proteins for degradation within cytoplasmic and nuclear compartments 36, 37. A set of fractionations followed by western blot experiments have revealed that proteasomes are present in the cytoplasm and in the nucleoplasm, while they are absent in the nucleolar and the nuclear envelope fraction 38. Another set of studies found certain proteasome subunits and subcomplexes have non-proteolytic functions in the nucleus 39, 40. More recent data in mammalian cells, including HCT-116 colon cancer cells, have shown that catalytically active proteasomes dominantly exist in the cytosol. These current data indicate that the majority of nuclear proteins are exported and degraded by the cytosolic proteasomes 41, and only a small portion of critical regulatory proteins, such as ribonucleoprotein complexes, are degraded by the proteasome complex inside the nucleus 42.

**Figure 3:**
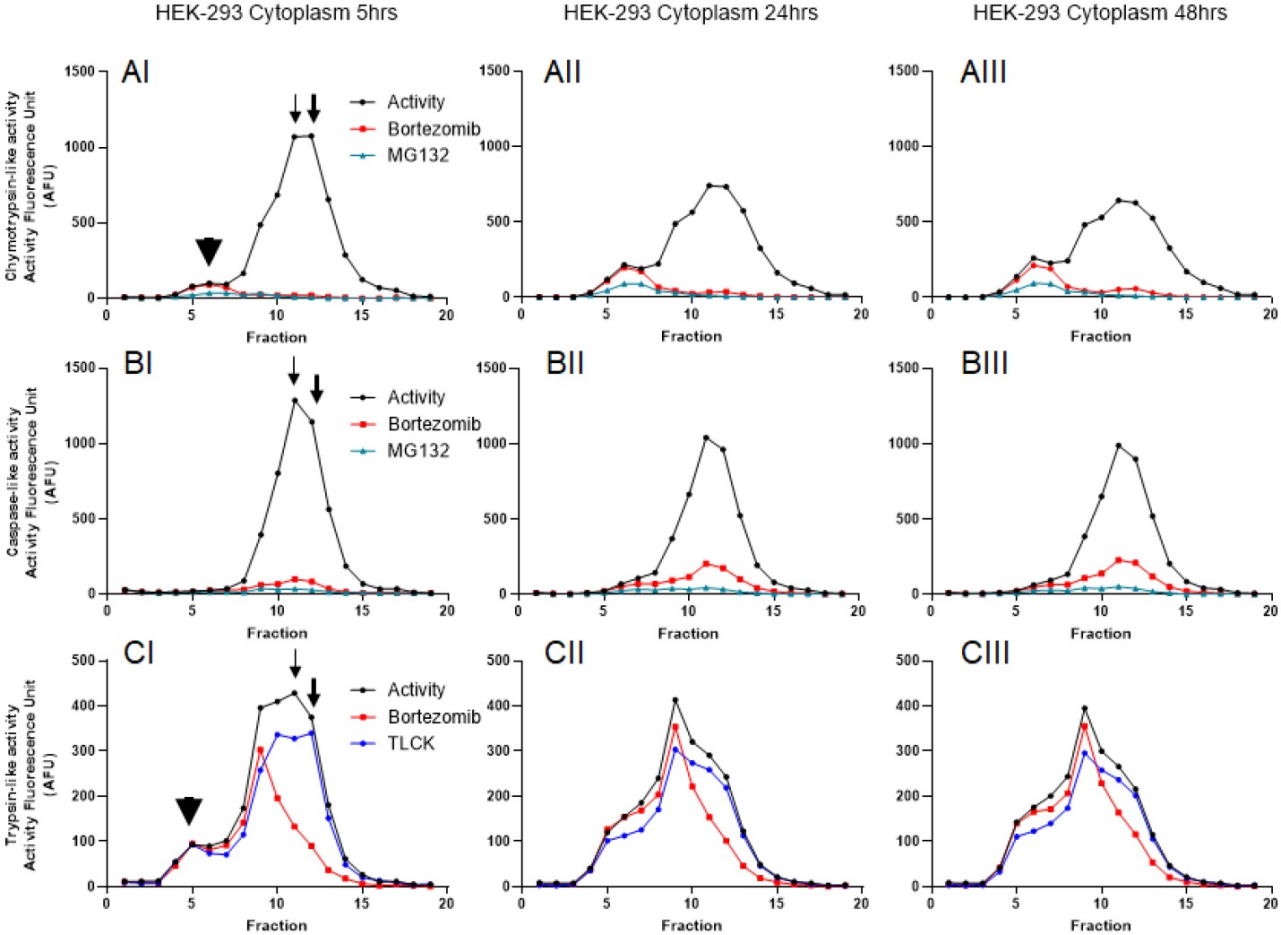
** Proteasome activity profile in HEK-293 cytoplasmic cell lysates subjected to iodixanol density gradient fractionation**. In each collected fraction, chymotrypsin- (A), caspase- (B), and trypsin-like (C) proteasome activities were measured in the presence or absence of proteasome inhibitors. To isolate the proteasome proteases' activities from other cellular proteases, measurements were repeated in the presence of three proteasome inhibitors: bortezomib (which targets the β5- and β1-subunit), MG132 (a nonspecific 20S proteasome inhibitor), or TLCK (a selective inhibitor of trypsin-like serine proteases). Arrowheads point to the potential 20S peaks (AI, CI; fraction 5) while thin and thick arrows show potential 26S and 30S proteasomes, respectively (AI, BI, CI; fractions 11-13). A proteasome activity profile was analyzed after 5 hours (I), 24 hours (II), and 48 hours (III) of incubation with AMC-labeled substrates in the presence of ATP. As expected, the addition of 2 mM ATP prevented 20S-19S dissociation. Experiments were performed in triplicate wells and repeated three times with similar results.

**Figure 4:**
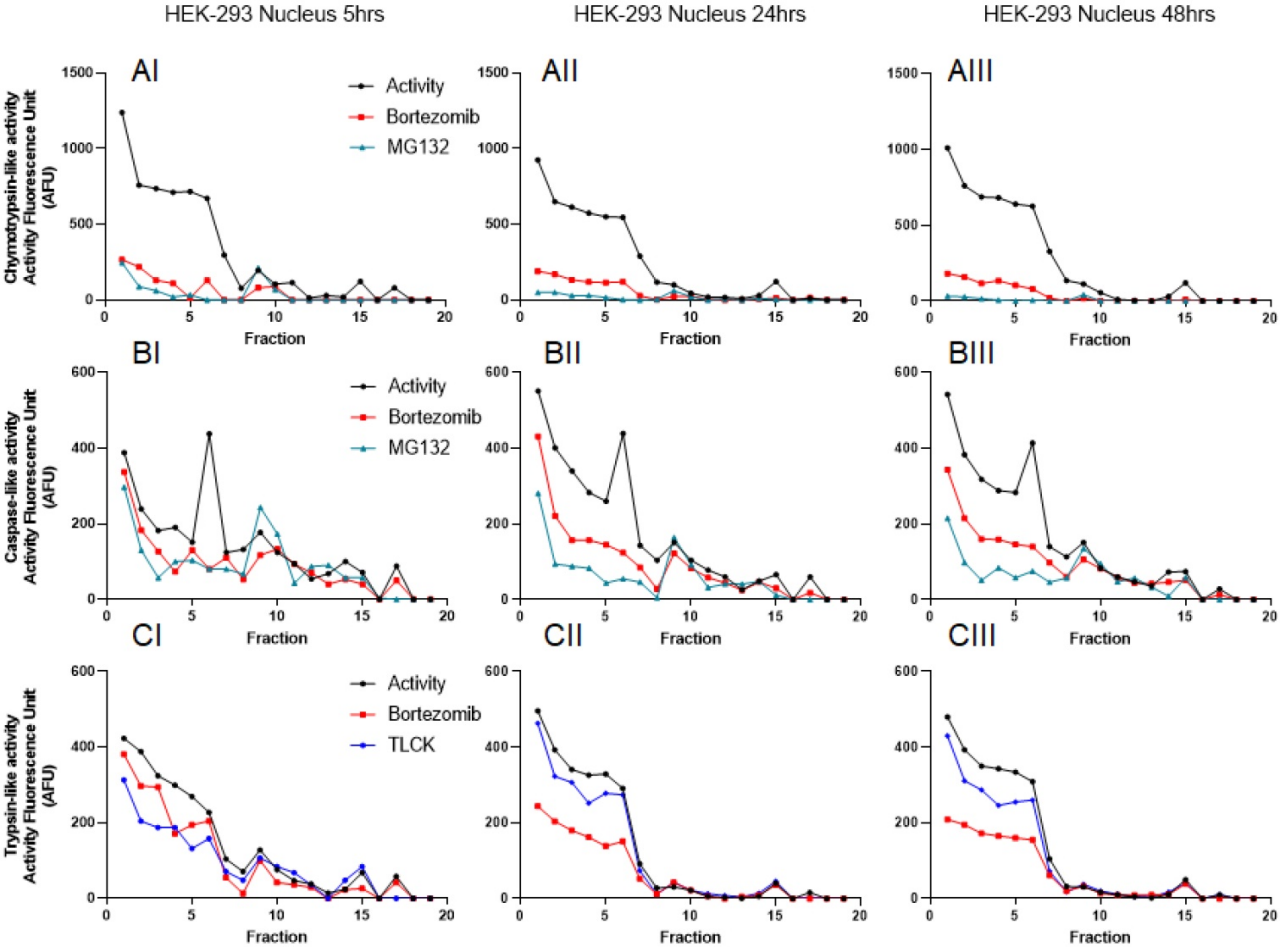
** Proteasome activity profile in HEK-293 nuclear cell lysates subjected to iodixanol density gradient fractionation.** The HEK-293 nuclear cell lysates were subjected to iodixanol density gradient fractionation. In each collected fraction, chymotrypsin- (A), caspase- (B), and trypsin-like (C) proteasome activities were measured in the presence or absence of proteasome inhibitors. To isolate the proteasome proteases' activities from other cellular proteases, measurements were repeated in the presence of three proteasome inhibitors: bortezomib, MG132, or TLCK. A proteasome activity profile was analyzed after 5 hours (I), 24 hours (II), and 48 hours (III) of incubation with AMC-labeled substrates in the presence of 2 mM ATP to maintain 26S and 30S integrity. While chymotrypsin-like activities showed early response to bortezomib and MG132, the trypsin-like and caspase-like activities were inhibited by proteasome inhibitors by a delay at 48hours incubation. Experiments were performed in triplicate wells and repeated three times with similar results.

To further understand the distribution of the proteasome complexes in the cytosol and nuclear lysates, we used iodixanol to fractionate both. An equal amount of total proteins was used for both the cytosol and the nuclear lysates, as previously suggested 41. Since fractionation experiments were conducted in the presence of 2 mM ATP, we were able to monitor catalytic activities of the proteasome for 48 hours (Figs. 3 and 4). Based on previous reports and linearity of fluorescent measurements over time, we decided to compare the results 5 hours after incubation with AMC-labeled substrates 38. Results show that all three proteasome activities are present in the cytosol and the nuclear compartments. However, the proteasome catalytic activities in the cytosol show the presence of potential 20S, 26S, and even some 30S 43 that migrated based on their densities around fractions 5 and 6 (20S; Fig. 3, Panels AI and CI, arrowheads) and fractions 11-13 (26S and 30S; Fig. 3, Panels AI, BI, and CI, thin and thick arrows, respectively). To verify that the recorded three catalytic activities are in fact proteasome catalytic activities, we used three types of proteasome inhibitors, as follows: 1) Bortezomib, an FDA-approved inhibitor, is a dipeptide boronic acid derivative and reversibly inhibits the β5-subunit and with a lower affinity inhibit the β1-subunit 16, 44; 2) MG132, which is a potent but nonspecific 20S proteasome inhibitor of all three activities 45; and 3) TLCK, which irreversibly inhibits trypsin-like serine proteases 46. These three proteasome inhibitors verified proteasomal catalytic activities measured in the HEK-293 cytosol lysates followed by iodixanol fractionation (Fig. 3, Panels A-C). The potential 20S catalytic activities (Fig. 3, Panels AI and CI, arrowheads) were inhibited by MG132 but had no response to bortezomib or TLCK. This latter observation suggests that the arrowhead peaks (Fig. 3, Panels AI and CI) may not be a pure 20S proteasome. On the other hand, the proteasome catalytic activities measured in nuclear fractions show a different distribution of proteasome complexes, which is due to their different densities and/or compositions (Fig. 4, Panels A-C). However, the proteasome inhibitors verified that these low-density proteasome complexes appear at the top of the gradient (fractions 1-5; Fig. 4, Panels AI-CI). Bortezomib and MG132 inhibitors successfully inhibited chymotrypsin-like activities in early fractions (Fig. 4, Panel AI), and their inhibition effectiveness stayed steady during 48hr incubation (Fig. 4, Panels AI-AIII). Bortezomib, MG132, and TLCK partially inhibited caspase-like and trypsin-like activities observed in the early fractions (fractions 1-5; Fig.4, Panels BI and CI). However, an extension of treatment with proteasome inhibitors allowed bortezomib to more effectively inhibit the proteasome complex present in fractions 1 through 5 (Fig. 4, Panels BII-BIII and CII-CIII). This lesser extent of catalytic activities in comparison to the chymotrypsin-like activity along a delayed kinetic has already been explained as occurring due to the alteration of proteasome complexes in response to proteasome inhibition 47. In addition, we found that while the levels of chymotrypsin-like (~1000 AFU) and trypsin-like (~400 AFU) activities of proteasome complexes are similar between the cytosol and the nuclear compartments, the caspase-activity of the nuclear proteasome is one-third of the corresponding activity in the cytosol (~400 AFU in Fig. 4, Panel BI, versus ~1200 AFU in Fig. 3, Panel BI). The activities of the proteasome in HEK-293 lysates were measured in three independent sets of experiments with similar results (Fig. S1).

### Proteasome complexes associated with cytoplasmic organelles

**Figure 5:**
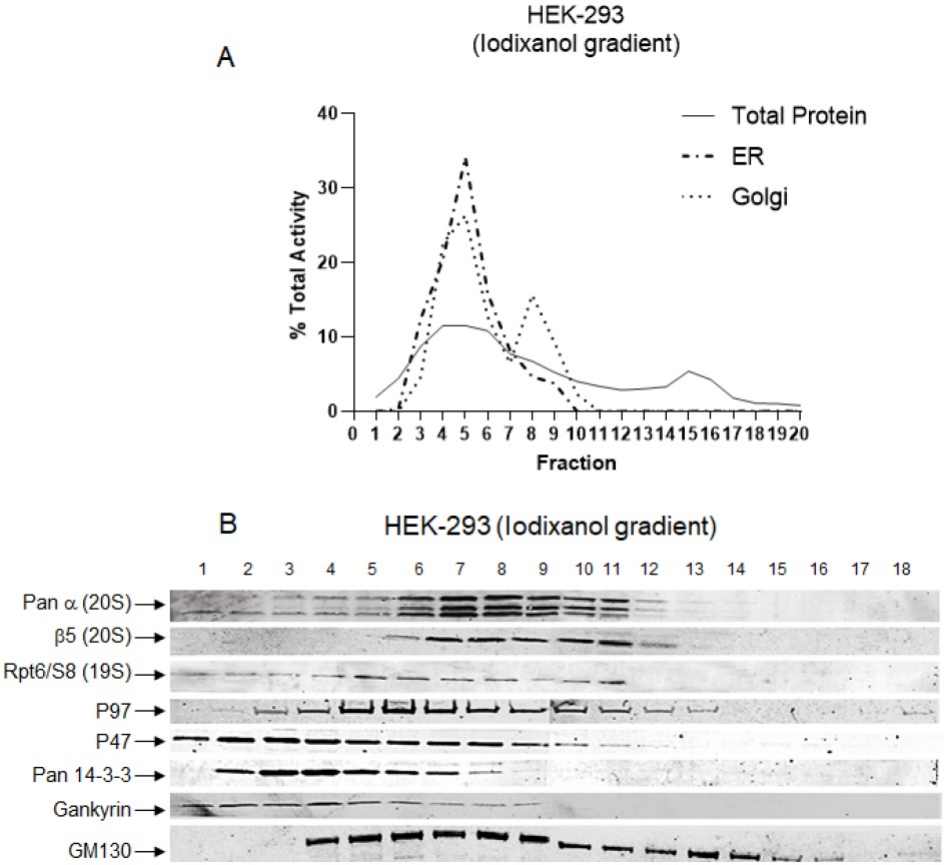
**The iodixanol gradient technique maintains physiological condition during isolation of proteasome subcomplexes.** The HEK-293 cytoplasmic cell lysates were subjected to an iodixanol density gradient fractionation with subsequent endoplasmic reticulum (ER) and Golgi apparatus assays (A) and western blot (B). (A) Analysis of ER and Golgi apparatus locations confirmed that iodixanol gradient fractionation efficiently isolates cellular compartments in their physiological conditions. (B) The results of western blot revealed that fractions with high proteasome activities are enriched with 20S core, 19S cap, and proteasome protein partners. The antibodies used were as follows: Anti-Pan-α, β5, and Rpt6/S8 ATPase antibodies to identify the presence of proteasome; p97, p47, and pan-14-3-3 antibodies as well as the cancer-specific proteasome-associated protein, Gankyrin, to determine the location of proteasome-associated proteins; and, finally, GM130 antibody to locate cis-Golgi. Experiments were performed in triplicate wells and repeated two separate times with similar results.

It has been shown that a specific set of proteasomes and their protein partners are associated with the ER, Golgi, and plasma membrane 48, 49. Based on the results obtained in Figures 3 and 4, we decided to determine the co-fractionation of the proteasome with the ER and Golgi in the iodixanol gradient fractionation. Assays for the presence of other subcellular compartments revealed that, in the HEK-293 cell line, the ER compartment had a peak in fraction 5, and the Golgi apparatus peaked in two distinct locations, fractions 5 and 8 (Fig. 5A). These results are consistent with the data of the expected ER and Golgi fractionation from the previous studies 23, 27. Using GM-130, we confirmed the presence of Golgi in fractions 4 to 14 (Fig. 5B). These results verified the integrity of the iodixanol gradient to separate ER and Golgi organelles based on their physiological densities. Western blot was also applied to confirm that the 20S core and 19S cap, as well as proteasome-associated protein partners, are present in fractions with high proteasome activities. In the HEK-293 cell line, we identified the presence of 20S catalytic core (Pan-ɑ and ꞵ5) and 19S regulatory complex (Rpt6-S8) in fractions 5 through 11 (Fig. 5B). These results are consistent with the proteasomal activity peaks in fractions 5 through 15 for chymotrypsin-, trypsin-, and caspase-like activity (Fig. 3). In addition, co-sedimentation of the 20S proteasome core subunits with a 19S subunit (Rpt6) suggests fractions 9-13 contains 20S proteasome complex with one or two 19S caps. Well-established proteasome-associated protein partners (p97, p47, pan 14-3-3) 50, 51 and the oncogenic proteasome-associated protein Gankyrin 52 were present in fractions with proteasomal activities (Fig. 5B). The potential proteasome 26S and 30S in Figure 3 and the location of the second peak of Golgi in Figure 5A suggest that a large part of capped proteasome complexes (26S and 30S) are co-fractionated with the Golgi apparatus. It has recently been shown that the 26S proteasome complex is associated with the Golgi apparatus as part of Golgi Apparatus-Related Degradation, or GARD, which plays a critical role in the late secretory pathway in normal conditions 48 and various secretion-related pathologies 53. Expectedly, the ERAD protein p97 54 co-fractionated with proteasome complexes present in enriched ER/Golgi compartments, while P47, pan 14-3-3, and Gankyrin were dominantly found in early fractions (fractions 1-6), which correspond to lighter forms of proteasome complexes. Association of Gankyrin with lighter proteasome complexes has been previously reported, since Gankyrin transiently associates with proteasome complexes to chaperone proteasome assembly 55.

### Diverse distribution of ER, Golgi, and proteasome complexes in colon, breast, and pancreas cancer cell lines

**Figure 6:**
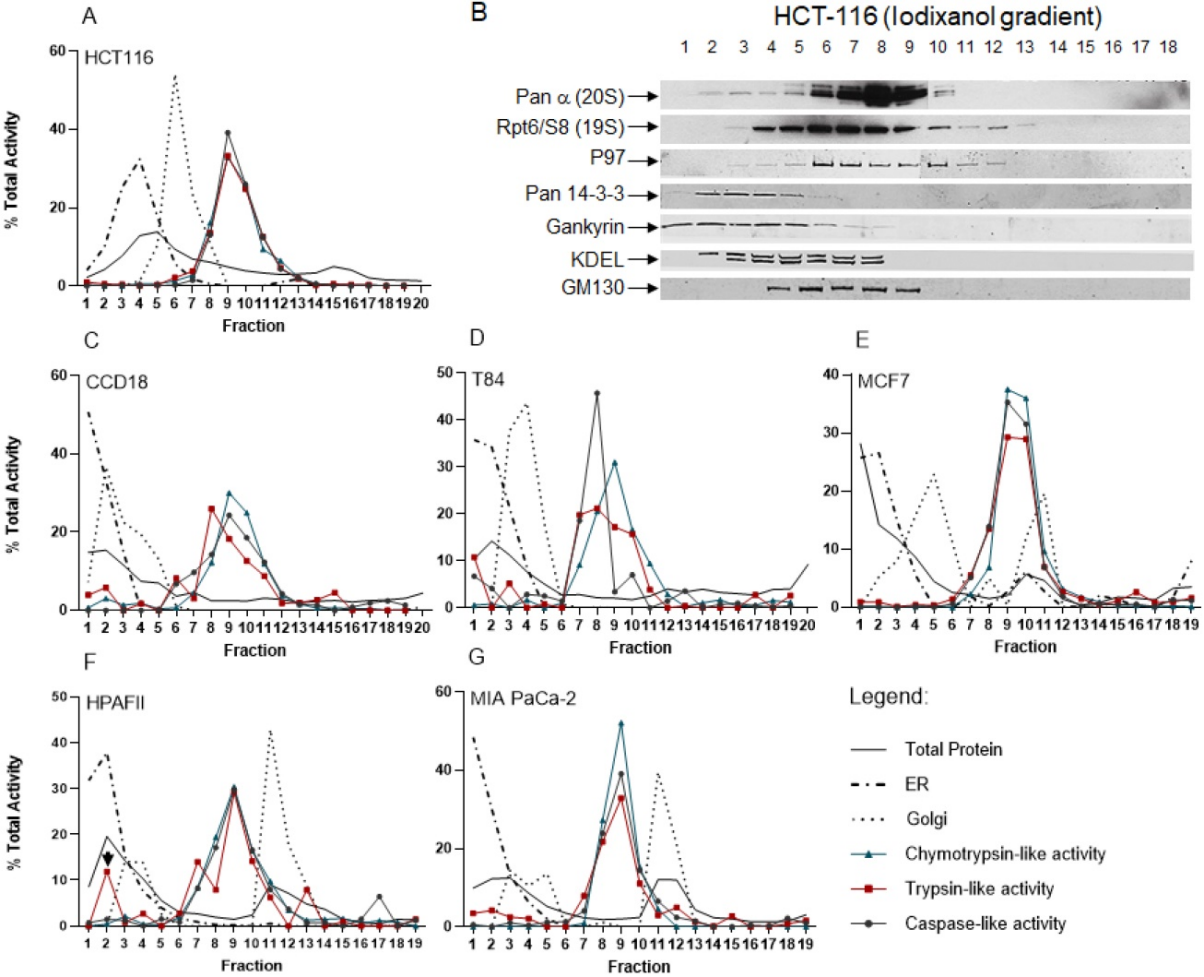
**Distribution of ER, Golgi, and proteasome complexes in colon, breast and pancreas cancer cell lines.** Normal colon fibroblast (C), colon (A, D), breast (E), and pancreas cancer cell lines (F, G) were screened for differences in sub-compartment sedimentation and proteasome activities. Equal volumes of cytoplasmic lysates obtained from each cell line were subjected to iodixanol gradient fractionation. Collected fractions were used for the ER and Golgi assays, BCA protein assay, and proteasome activity analysis. (B) Western blots were conducted on collected fractions after iodixanol gradient fractionation of HCT-116 cytoplasmic cell lysates. The results confirm that the 20S core, 19S cap, and proteasome-associated protein partners are presented in the fractions with high proteasomal activities. Pan-α and Rpt6/S8 antibodies identified the presence of proteasome complexes. P97 and pan-14-3-3 as well as Gankyrin (a cancer-specific proteasome-associated protein) antibodies represent proteins associated with proteasome complexes. The KDEL and cis-Golgi (GM130) antibodies determined locations of ER and Golgi compartments, respectively. Experiments were performed in triplicate wells and repeated two separate times with similar results.

After establishing the iodixanol gradient fractionation method in HEK-293 cells, we used the iodixanol gradient technique to fractionate cytoplasmic lysates obtained from normal colon fibroblast CCD-18, HCT-116, and T84 colon cancer cell lines as well as breast cancer cells (MCF7) and two pancreas cancer cell clines (HPAFII and MIA PaCa-2). In the HCT-116 colon cancer cell line, we first determined the locations of the ER and Golgi apparatus. The ER and Golgi assays revealed a separation of the two subcellular compartments with two peaks in fractions 4 and 6, respectively (Fig. 6A). We also used a BSA assay and total protein staining to determine the protein distribution in all 20 collected fractions in HCT-116 cells (Figs. 6A and S2). The proteasomal catalytic activity showed a consistent pattern for all three active sites peaking in fractions 8 through 11 (Fig. 6A). Western blot results confirmed that the 20S core and 19S cap, as well as the proteasome-associated protein partners (p97, Pan 14-3-3, and Gankyrin), are presented in the fractions with high proteasomal activity (Fig. 6B). Anti-KDEL (ER marker) and anti-GM130 (Golgi marker) antibodies were used to locate ER and Golgi compartments. Interestingly, the ER and Golgi showed different distribution profiles in CCD-18 normal fibroblast cells as well as T84 derived from a colon cancer metastasis in the lung (Fig. 6, Panels C and D). However, iodixanol gradient fractions showed no major differences in terms of the three catalytic proteasome activities in CCD-18 non-cancerous cells versus the HCT-116 and T84 colon cancer cell lines. We observed a similar early ER distribution profile in MCF7 breast cancer cells as well as the HPAFII and MIA PaCa-2 pancreas cancer cell lines. However, the Golgi compartments were different in these three cancer cell lines, demonstrating two distinct peaks (Fig. 6, Panels E-G). While these three cell lines (Fig. 6, Panels E-G) show a similar distribution of proteasome catalytic activities, we observed a distinguished trypsin-like catalytic activity in the HPAFII cells (Fig. 6F, arrowhead) in low-density fractions. It has been reported that the presence of an intermediate proteasome that is constitutively expressed occurs in pancreatic beta cells, and they respond to stimulatory induction by interleukin 1β 56. The proteolytic activities in Figure 6 were illustrated as percentages (see “Materials and Methods” section).

### Proteasomal catalytic assays indicate a set of subclasses of proteasome complexes among normal and cancerous colon cell lines

**Figure 7:**
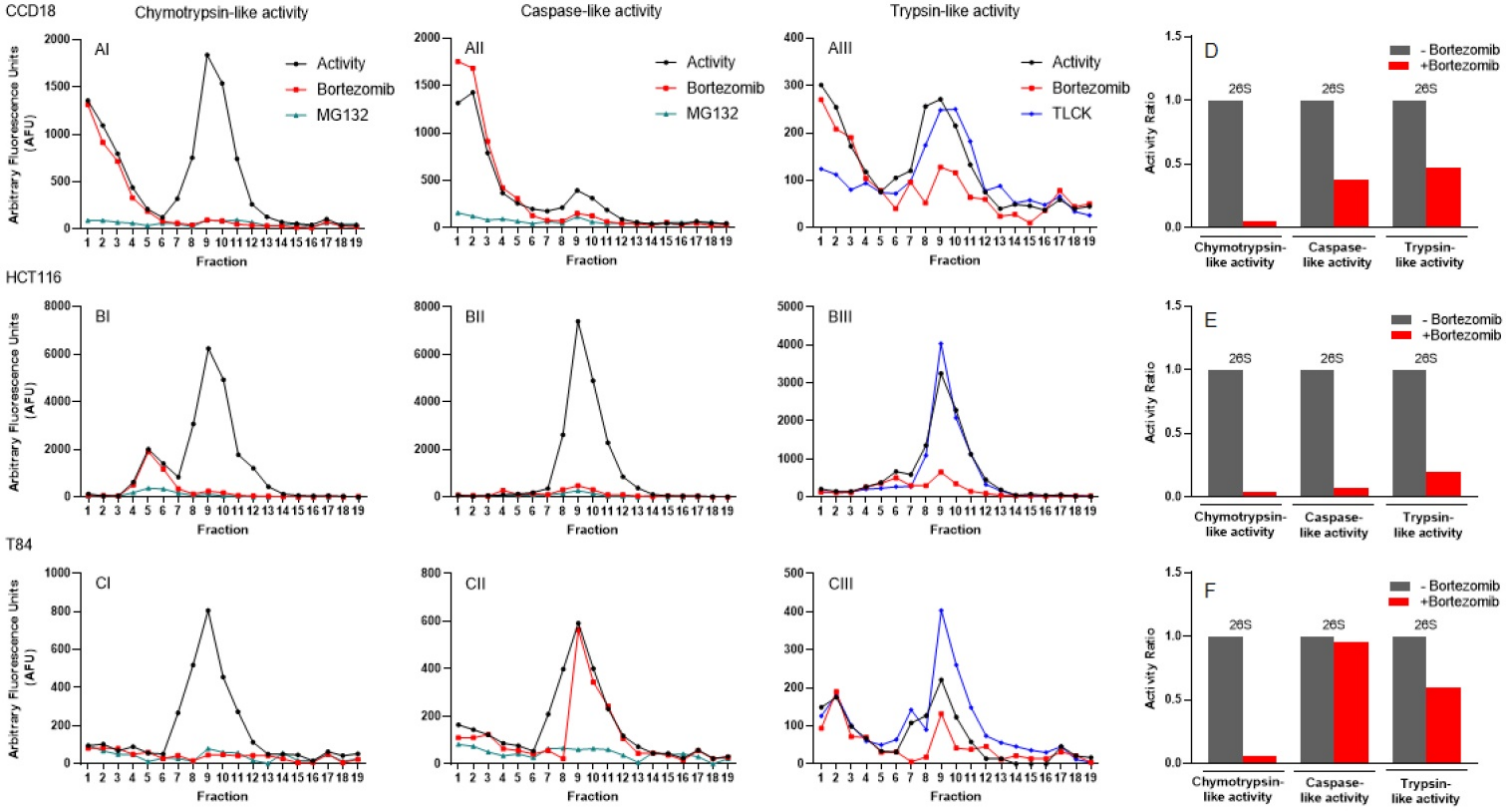
**Proteasomal catalytic activities of normal and cancerous colon cell lines subjected to iodixanol gradient fractionations indicate proteasome heterogeneity**. (A-C) Three proteasome catalytic activities were identified under normal conditions and after incubation with three proteasome inhibitors. Chymotrypsin- (I), caspase- (II), and trypsin-like (III) proteasome activities were measured in the line of normal colon fibroblasts (A) and colon cancer cell lines (B, C) before and after application of bortezomib and MG132 or TLCK. (D-F) Bar graphs show the ratio of three 26S proteasome activities without and with the proteasome inhibitor bortezomib. Experiments were performed in triplicate wells and repeated two separate times with similar results.

Once the methodology was developed and assessed in a non-cancerous control cell line, HEK-293 (Figs. 3 and S1), and in a commonly-utilized colon cancer cell line, HCT116 (Fig. 6), collected fractions from colon, breast, and pancreatic cancer cells were treated with proteasome inhibitors. In CCD-18 normal colon fibroblast cells, we had a set of early enzymatic activities that were inhibited by MG132 but not bortezomib (Fig. 7, Panels AI-AIII). The second set of peaks located in fractions 8-11 (Fig. 7, Panels AI-AIII) were confirmed as the 26S complex, since it is inhibited by bortezomib as well as MG132. As expected, chymotrypsin-like activity showed the maximum inhibition by bortezomib, and caspase and trypsin showed modest inhibition in CCD-18 cells (Fig. 7, Panel D). The proteasome activities in HCT-116 cells with and without proteasome inhibitor (Fig. 7, Panels BI-BIII) similarly showed dominant 26S between fractions 8 and 11, similar to CCD-18 non-cancerous cells. However, there were three differences: 1) A 20S-like peak (fractions 5-6) was only inhibited by MG132; 2) The level of 26S proteasome activities was remarkably higher in HCT-116; they were 4 times higher for chymotrypsin-like (Fig. 7, Panel BI versus AI) and caspase-like (Fig. 7, Panel BII versus AII) activities as well as 12.5 times more for trypsin-like activities (Fig. 7, Panel BIII versus AIII); and 3) Interestingly, bortezomib remarkably (>90%) suppressed all three activities of the proteasome in HCT-116 in comparison to CCD-18 normal colon cells (Fig. 7, Panel E versus D); this might be due to the elevated proteasome sensitivity to inhibitors under chronic stress observed in cancer cells, as previously described in multiple myeloma 57. In the T84 colon cancer cell line, the three proteasome activities had a major peak between fractions 7 and 11 (Fig. 7, Panels CI-CIII). While the 26S proteasome completely responded to bortezomib (Fig. 7, Panel CI), the caspase-like and trypsin-like activities had only partial inhibition in the presence of bortezomib (Fig. 7, Panels CII, CIII, and F). Surprisingly, TLCK failed to suppress trypsin-like activities in all three cell lines.

### Diverse cancer cell lines have shown variation in response to common proteasome inhibitors

**Figure 8:**
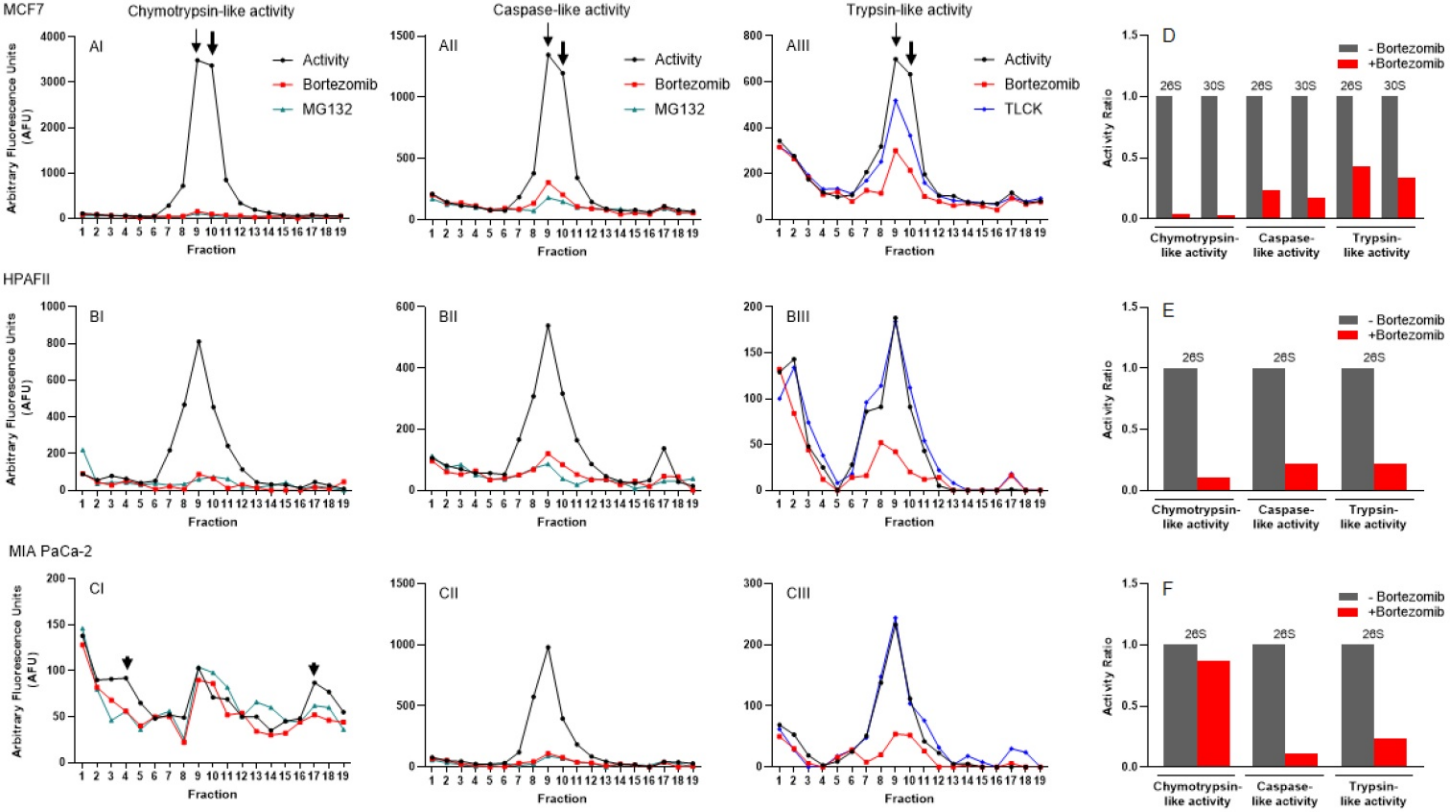
** Proteasomal catalytic activities measured in breast and pancreatic cancer cell lines indicate proteasome heterogeneity induced by different oncogenic drivers.** (A-B) Three proteasome activities were identified under normal conditions and after introduction of a selected proteasome inhibitor. Chymotrypsin- (I), caspase- (II), and trypsin-like (III) proteasome activities were measured in breast (A) and pancreatic (B, C) cancer cell lines in the absence and the presence of bortezomib and MG132 or TLCK. Two arrows, one thin and one thick, point to fractions 9 and 10, corresponding to potential 26S and 30S in MCF7 cells, respectively. (D-F) Bar graphs show the ratio of three potential 26S and 30S proteasome catalytic activities without and with the proteasome inhibitor bortezomib. Experiments were performed in triplicate wells and repeated two separate times with similar results.

In addition to colon cancer cell lines, we used the same approach to determine the response of proteasome complexes to proteasome inhibitors in breast and pancreas cancer cell lines. In the MCF7 breast cancer cell line, we observed a dominant proteasomal catalytic activity in fractions 8 to 11, which was suppressed remarkably by both bortezomib and MG132 proteasome inhibitors (Fig. 8A). As previously described 43, we observed the presence of two heavy peaks at fractions 9 and 10, respectively, corresponding to potential 26S and 30S in MCF7 cells (Fig. 8, Panels AI-AIII; arrows). Ongoing experiments in our lab will determine the composition of 26S and 30S recorded in Fig 8AI-III. Panel D in Figure 8 shows the suppression of three catalytic activities in isolated proteasomes in response to bortezomib in MCF7 cells. In contrast to colon cancer cells, trypsin-like activity partially responded to TLCK in MCF7 cells. In the HPAFII pancreatic cancer cell line, the measured proteasomal catalytic activity showed a single peak at fraction 9 for all three catalytic activities (Fig. 8, Panels BI-BIII). There were two unique patterns recorded in HPAFII in comparison to the other cancer cell lines: 1) The trypsin-like activity showed an early peak (fractions 1-3) that had no response to bortezomib and TLCK, and 2) Similar to HCT-116, the three 26S catalytic activities were dominantly suppressed by bortezomib in HPAFII cells (Fig. 8E). Finally, the MIA PaCa-2 pancreatic cancer cell line showed the usual single proteasome peak for caspase-like and trypsin-like activities (Fig. 8, Panels CII and CIII). However, chymotrypsin-like activities appeared with three non-organized peaks. Based on the typical density of 26S, we considered the individual peak at fraction 9 to be the potential 26S proteasome. Surprisingly, this peak showed a low inhibition response to bortezomib (Fig. 8, Panels CI and F). However, the other lighter peak (fractions 3 and 4; likely 20S) and heavier peaks (fractions 17 and 18; likely 30S) responded to both bortezomib and MG132 (Fig. 8CI; arrowheads). More studies need to be done to determine whether these peaks are in fact corresponding to the proteasomes in the MIA PaCa-2 cell line. The proteasomal activities in Figures 7 and 8 were recorded 5 hours after incubation with proteasome inhibitors in the presence of 2 mM ATP.

The average inhibition of the three catalytic activities of the 26S proteasome by the proteasome inhibitors bortezomib, MG132, or TLCK measured in control (HEK-293), colon cancer (HCT-116 and T84), normal colon fibroblast (CCD-18), breast cancer (MCF7), and pancreatic cancer (HPAFII and MIA PaCa-2) cell lines is summarized in Table 1. The measurements taken in the presence of the inhibitors were compared with proteasomal catalytic activities in normal conditions (no proteasome inhibitor, bortezomib), and the percent of the inhibition was calculated. The percent of activity reduction remarkably varied for chymotrypsin-, trypsin-, and caspase-like catalytic activities in individual cell lines in a tissue-specific manner.

## Discussion

An elevated level of proteasome subunit expression and higher proteasome activity was observed in several types of solid tumors 58, 59. These hyperactive proteasome complexes support cancer cells by maintaining cell survival, cell cycle, and cell differentiation 60. These findings accelerate continuing interest in expanding the application of proteasome inhibitors that have been successfully implemented for the treatment of hematological cancers 61-63. However, the clinical efficacy of the first-in-class proteasome inhibitor, bortezomib, was limited in solid cancers 17. The second generation of agents targeting the UPS also had significant limitations, including a narrow therapeutic window against solid tumors and the development of drug resistance 16, 64. Although the efficacy of the proteasome inhibitors can be improved by use in combination with traditional chemotherapeutic agents, this approach is associated with a list of adverse effects 65.

While targeting hyperactivated proteasomes in solid tumors has a potential therapeutic benefit, the compensatory mechanisms activated in response to proteasome inhibition, such as activation of autophagy and prosurvival pathways as well as the elevation of proteasome expression and generation of mutations in beta-subunit genes, can neutralize the anti-tumorigenic function of proteasome inhibitors in solid tumors 66, 67. Moreover, well-established evidence indicates that low proteasome activity in cancer stem cells in diverse solid tumors can reverse the sensitivity of tumors to proteasome inhibitors 68-72. Finally, it has been reported that inhibition of β2 and β5 subunits of 20S proteasome can activate epithelial-mesenchymal transition (EMT), which is associated with the formation of cancer stem cells and induction of the metastatic cascade 73. In fact, inhibition of the proteasome in immortalized human mammary epithelial cells leads to self-renewal capability and cancer stem cell features 73. Together, the above evidence indicates proteasome inhibitors can lead to different outputs based on tumor heterogeneity, type, and stage.

Besides intra-tumor heterogeneity, a complex set of post-translational modifications and the intrinsic structural diversity of subunits in hybrid proteasome complexes as well as proteasome regulators and partners significantly contribute to diverse forms of proteasomes in cancer cells 74, 75. Interestingly, the presence of gender-related heterogeneity of proteasome forms has been reported in animal models 76. Finally, differences in proteasome activities between nuclear and cytoplasmic fractions show another level of proteasome diversity 77. Thus, further exploration of proteasome heterogeneity and related resistance determinants, especially in solid tumors, is required for the development of more effective anticancer therapeutics based on UPS targeting.

To understand the concept of proteasomal heterogeneity, in the present study we compared proteasome complexes among three different originated cancer cell lines using an iodixanol gradient fractionation technique. In contrast to traditional methods such as glycerol and sucrose, the iodixanol chemical features allow maintenance of a constant iso-osmotic environment throughout the density gradient and provide a more efficient distribution of protein complexes based on their sizes and structural compositions (Figs. 1 and 2). Separation and subsequent centrifugation of cytoplasmic proteins were based on evidence of proteasome differences between cytoplasmic and nuclear compartments (Figs. 3 and 4). The iodixanol method allows enrichment of proteasomes complexes in their native structure with their associated protein complexes or cytoplasmic compartment, such as ER and Golgi (Fig. 5). Physiologically isolated proteasomes complexes allow further analysis of enriched proteasomal subpopulations in a tissue- and cancer-specific dependent manner.

Applying the improved method of proteasome isolation, we contrasted proteasome catalytic activity and subcellular compartment distribution in both normal and cancer cell lines. The results illustrated in Figure 6 indicate the difference in the ER and Golgi compartments' distributions among colon, breast, and pancreatic cancer cell lines. In breast and pancreatic cancer cell lines, ER and Golgi compartments appeared as two distinct activity peaks, the earlier of which matched with the peak indicated in normal and colon cancer cell lines. These differences are likely due to the presence of an advanced trans-Golgi network (TGN) in breast and pancreas cell lines 78. Besides, co-sedimentation of ER and Golgi apparatus with proteasomes differs markedly among the three cancer cell lines (Fig. 7).

A large number of published studies have shown that the proteasome degradation machinery maintains a dynamic crosstalk with ER and Golgi apparatus as part of endoplasmic-reticulum associated degradation (ERAD) and the Golgi Apparatus-Related Degradation (GARD) pathways 79. The iodixanol gradient fractionation experiments in this study showed a subclass of proteasome complexes co-sediment with ER or Golgi. The evaluation of ER and Golgi localization and their associated proteasome complexes as part of ERAD or GARD can help in assessing changes within these subcellular compartments in cancer cells treated with proteasome inhibitors with and without chemotherapies. In addition to enzymatic assays, we used western blot technique to verify the location of the ER and Golgi, plus some known proteasome partners such as Gankyrin. The iodixanol gradient fractionation allows the relation of these protein partners 80 with the proteasome complexes in the presence of proteasome inhibitors to be determined. In comparison to the resolution of sucrose or glycerol sedimentation, this current study suggests that the iodixanol density gradient fractionation may lead to the isolation of substantially pure individual proteasome subcomplexes in a sub-cellular compartment-dependent manner. Proteomic assays can determine unique stable or transient partners associated with these proteasome subcomplexes. More importantly, future studies will determine the structural properties (20S, 26S and 30S proteasomes) and the sensitivity of these isolated proteasome subcomplexes to current and new proteasome inhibitors developed by scientists.

To characterize the sensitivity of cancer cells to proteasome inhibitors, we analyzed proteasome activity profiles under normal conditions and in the presence of bortezomib, MG132, or TLCK proteasome inhibitors. As previously described, the proteasome complexes migrated based on their sizes, from the lightest form, the 20S particle, to the 26S (singly capped with one 19S regulator subunit) and finally the 30S (doubly capped) proteasome 26. Regardless of cell types, three proteasomal catalytic activities shared the position of the potential main 26S proteasome complex; however, pancreatic cancer and colon cancer cell lines showed several proteasomal subcomplexes, represented by additional peaks within both lower and higher density fractions. The activity profiles with introduced proteasome inhibitors show the differences in responses to bortezomib, MG132, or TLCK among colon, breast, and pancreatic cancer cell lines (Figs. 7 and 8 and Table 1).

As previously described 67, observed variations in sensitivity to proteasome inhibitors among different types of solid cancers can be caused by the existence of proteasomal heterogeneity and regulators, which are affected by different oncogenic drivers and drug-resistance mechanisms developed in a cancer-dependent manner. Therefore, the isolation of heterogeneous proteasome complexes in their native forms is critical to measure the effectiveness of novel proteasome inhibitors in the targeted cancer tissues.

Together, these results provide a unique profile of proteasomal catalytic activity and subcellular compartment localization in colon, breast, and pancreatic cancer cell lines. This developed methodology has shown efficacy in the isolation of proteasome complexes maintained in their physiological conditions. Thus, the data presented in the study can be a valuable guidance for the study of proteasome heterogeneity between cancer cell lines and its association with the effectiveness of novel proteasome inhibitors and other types of anticancer therapeutics. Our future studies will investigate proteasomal activities in human tumor tissues using above iodixanol gradient fractionation.

## Supplementary Material

Supplementary method, figures and tables.

## Figures and Tables

**Table 1:** Proteasome catalytic activity suppression in response to proteasome inhibitors indicates proteasome heterogeneity in a cancer-dependent manner

	Percent Inhibited					
	Chymotrypsin-like activity		Caspase-like activity		Trypsin-like activity	
	Bortezomib	MG132	Bortezomib	MG132	Bortezomib	TLCK
HEK-293	93	94	68	78	61	2
CCD-18	93	93	62	74	54	4
HCT-116	94	97	93	96	80	0
T84	92	90	33	83	63	-64
MCF7	95	97	69	73	60	22
HPAFII	91	88	75	82	72	-12
MIA PaCa-2	12	-7	87	89	72	-8

The table represents the average activity reduction of three catalytic activities by proteasome inhibitors bortezomib and MG132 or TLCK in control (HEK-293), normal colon fibroblast (CCD-18), colon cancer (HCT-116 and T84), breast cancer (MCF7), and pancreatic cancer (HPAFII and MIA PaCa-2) cell lines.
