# Supplementary material for: Proteasome Complexes and Their Heterogeneity in Colorectal, Breast and Pancreatic Cancers
Source: J Cancer. 2021 Mar 5;12(9):2472–87. doi: 10.7150/jca.52414 (PMC8040722; doi:10.7150/jca.52414)
Supplement: Supplementary file 1 — Supplementary method, figures and tables. [file jcav12p2472s1.pdf]

Iodixanol Gradient Analysis

This protocol is a modified version of an iodixanol gradient instruction available in http://www.axis-shield-density-gradient-media.com/ site.

1. Prepare the following stock solutions:
   a. HEPES stock (free acid, from 4ºC fridge) = 11.9g per 100ml water
   b. EDTA stock ($Na_2 \cdot 2H_2O$, from 4ºC fridge) = 3.72g per 100ml water
2. Prepare Homogenization Medium (**HM**) as follows:
   a. Use pre-made from 4ºC or make by dissolving 17g sucrose in 100ml water, add 2ml of EDTA stock and 4ml of HEPES stock and adjust to pH 7.4 with 1M NaOH.
3. Prepare Working Solution (**WS**) as follows:
   a. The diluent solution: use pre-made from 4ºC or make by dissolving 8.5g sucrose in 50ml water, add 6ml EDTA stock and 12ml HEPES stock and adjust to pH 7.4 with 1M NaOH.
   b. Per 4 gradient tubes mix 25ml Optiprep and 5 ml of the diluent solution.
   c. Per 2 gradient tubes mix 12.5ml Optiprep and 2.5ml of the diluent solution.
4. Create 4 concentrations of WS and HM in 50ml tubes as follows:

	Per 4 tubes		Per 2 tubes	
% iodixanol	WS (ml)	HM (ml)	WS (ml)	HM (ml)
8	2.00	10.50	1.00	5.25
16	4.00	8.50	2.00	4.25
28	7.00	5.50	3.50	2.75
38	9.50	3.00	4.75	1.50

5. Layer 2.5 ml of each gradient using Auto DensiFlow Probe with 38% at bottom (first), then 28%, 16% and 8%. You can measure 2.5ml into separate tubes, then feed into probe. Store the remaining made solutions in 4°C fridge until the next day if you need to repeat.
6. Layer ~1ml of cell lysate at the top of the preformed, precooled Iodixanol gradient.
7. Centrifuge at 28500rpm (100,000xg) for 18hours using a pre-chilled Beckman SW41Ti rotor at 4ºC. Caution: Avoid disturbing the pre-made gradients before placing in the rotor.
8. Collect 20 equal volume fractions (500µl each) starting from the top of the tube using Auto DensiFlow gradient collector.
9. To preserve the integrity of 20S and 30S assembly, all samples must be kept cold at 4°C during handling and centrifugation.
10. Keep the samples at -20 for future protein and enzyme analysis.

Cancer cell line	Tumor types	Age	Gender	Reference
HCT-116	colorectal carcinoma	Adult	Male	PMID: 7214343
T84	colorectal carcinoma	72 years	Male	PMID: 8794293
MCF7	adenocarcinoma	69 years adult	Female	PMID: 4357757
HPAFII	adenocarcinoma	44 years	Male	PMID: 2734279
MIA PaCa-2	carcinoma	65 years	Male	PMID: 7558455

Table S1: Human cancer cell lines used in this study.

Ultra-gradient fractionation	Type	Reference
100,000 g for 22 h	Glycerol	PMID: 21640720
27,000 rpm for 20 h	Iodixanol	PMID: 29074393
38 000 rpm for 16 h	Glycerol	PMID: 9119074
38 000 rpm for 16 h	Glycerol	PMID: 11854272
25,000 rpm for 22 h	Glycerol	PMID: 10490597
174,000 g for 12 h	Sucrose	PMID: 23727017
83,000 g for 22 h	Glycerol	PMID: 23029399
150,000 g × g 21 h	Sucrose	PMID: 25367127
48,000 rpm for 4 h	Glycerol	PMID: 20682791
29,000 rpm for 12 h	Sucrose	PMID: 10438810
100,000 g for 18 h	Sucrose	PMID: 17189251
82,200g for 22 h	Glycerol	PMID: 20674016

Table S2: A list of articles which reported the presence of proteasome heterogeneity in cells using glycerol- or sucrose-based gradient fractionation.

Figure 1-Supplement: **Proteasome activities in HEK-293 lysates in three independent sets of experiments**. Chymotrypsin- (A), caspase- (B), and trypsin-like (C) proteasome activities measured in twenty fractions and collected following iodixanol gradient fractionations of HEK-293 cytoplasmic cell lysates. The similar results in each set confirm the repeatability of the method (n=3).

Figure 2-Supplement: **Measurement of protein migration of HCT-116 cytoplasmic cell lysates in iodixanol gradient fractions**. (A) The BCA assay shows the distribution of total protein in fractions after application of the iodixanol gradient fractionation. (B) SYPRO Ruby

protein gel staining (ThermoFisher) of 20 fractions collected in the iodixanol gradient fractionation of the HCT-116 cytoplasmic cell lysates. This ready-to-use fluorescent method detects total proteins in multiple bands separated by polyacrylamide gel electrophoresis. The results confirm that peaks in fractions 2-5 and 12-14 correspond to two protein peaks in the SYPRO staining. Ongoing proteomic approach in our lab will determine enriched proteins in these two peaks and their relations to proteasome complexes sedimented in these two peaks.

Fig. S1

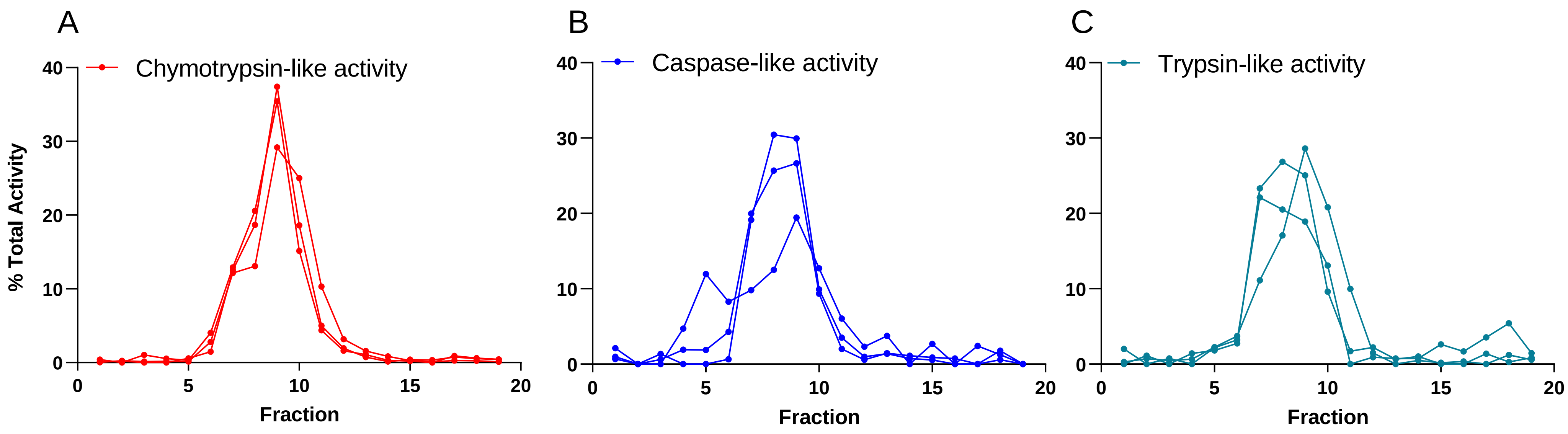
A
Chymotrypsin-like activity
% Total Activity
Fraction
B
Caspase-like activity
Fraction
C
Trypsin-like activity
Fraction

Fig. S2

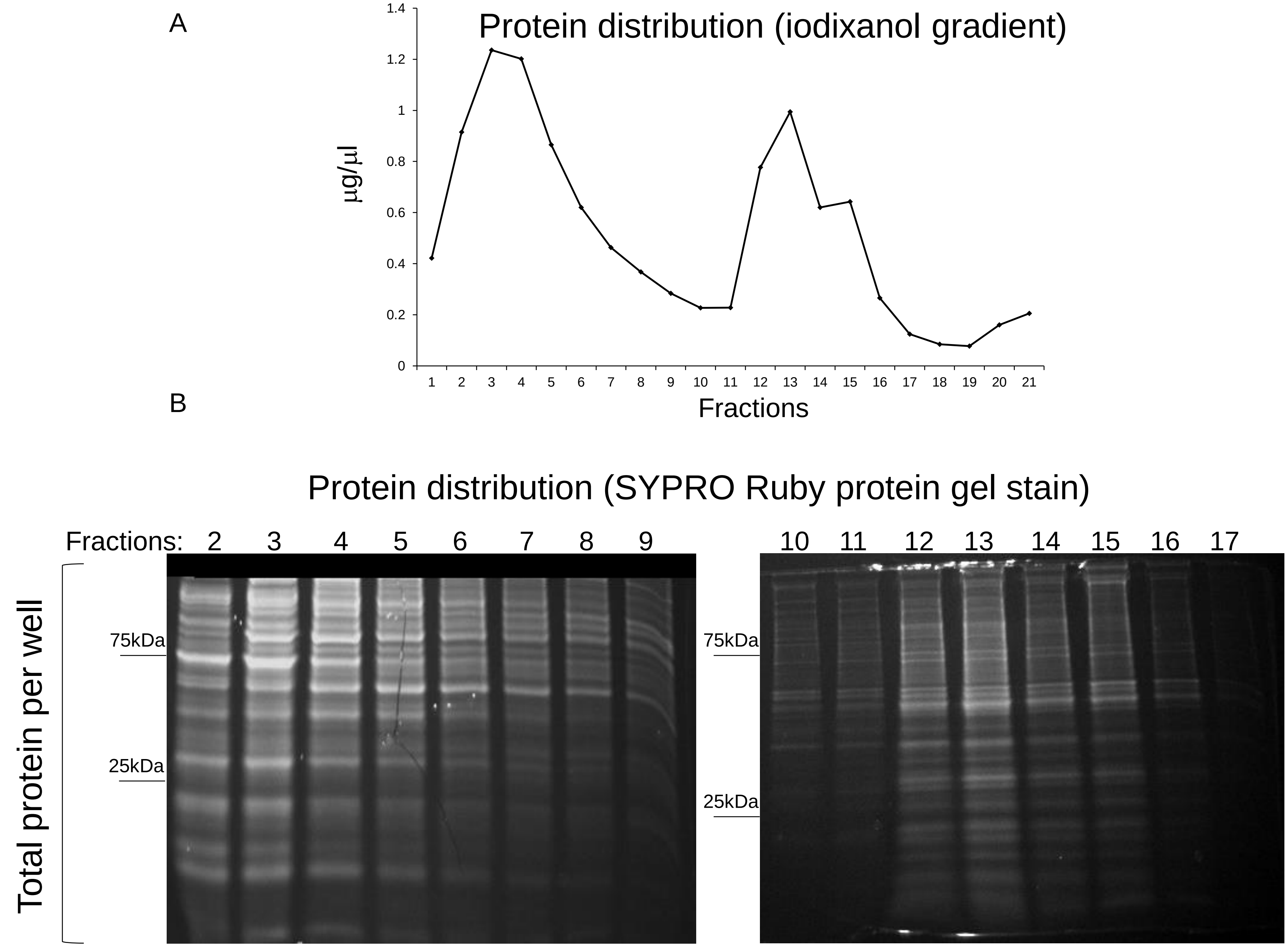
A
HCT-116 cells
Protein distribution (iodixanol gradient)
μg/μl
1.4
1.2
1
0.8
0.6
0.4
0.2
0
1 2 3 4 5 6 7 8 9 10 11 12 13 14 15 16 17 18 19 20 21
Fractions
B
Protein distribution (SYPRO Ruby protein gel stain)
Fractions: 2 3 4 5 6 7 8 9
10 11 12 13 14 15 16 17
Total protein per well
75kDa
25kDa
75kDa
25kDa
